# 

**Published:** 1896-11

**Authors:** 


### TABLE OF CONTENTS.

ORIGINAL COMMUNICATIONS. PAGE

Certain Manifestations of Syphilis of Importance in the Practice of Dentistry. By CHARLES M. WHITNEY, Boston, Mass. . . . . . . . . 685
Electricity and its Applications in Dentistry. By WILLIAM E. CLARK, S.B., and PHILIP W. DAVIS, A.B., S.B. . . . . . . . . . . . . . . . 694
Cataphoresis. By HENRY W. GILLETT, D.M.D. . . . . . . . . . . . . . 704
Porcelain Fillings. By ELOF FÖRBERG, Stockholm, Sweden . . . . . . . 713
Pyorrhœa Alveolaris. By DR. EUGENE S. TALBOT, Chicago, Ill. . . . . . . 717

ABSTRACTS AND TRANSLATIONS.

Report on a Case of Reunited Fractured Human Tooth. By MR. STORER BENNETT. . . . . . . . . . . . . . . . . . . . . . . . . . . . . 718

REPORTS OF SOCIETY MEETINGS.

American Dental Association . . . . . . . . . . . . . . . . . . . . . 720
American Academy of Dental Science . . . . . . . . . . . . . . . . . 737
Odontological Society of Pennsylvania . . . . . . . . . . . . . . . . 747

EDITORIAL.

Examining Boards and their Selection . . . . . . . . . . . . . . . . 749
Dr. Williams's Retort . . . . . . . . . . . . . . . . . . . . . . . . 753

BIBLIOGRAPHY . . . . . . . . . . . . . . . . . . . . . . . . . . . . 756

OBITUARY.

Dr. Edwin A. Stebbins . . . . . . . . . . . . . . . . . . . . . . . . 762

CURRENT NEWS.

Philadelphia County Dental Society . . . . . . . . . . . . . . . . . . 763

SELECTIONS.

The Value of Antipyretics . . . . . . . . . . . . . . . . . . . . . . 764

